# Neodymium as an alternative contrast for uranium in electron microscopy

**DOI:** 10.1007/s00418-020-01846-0

**Published:** 2020-02-01

**Authors:** Jeroen Kuipers, Ben N. G. Giepmans

**Affiliations:** Department of Biomedical Sciences of Cells and Systems, University of Groningen, University Medical Center Groningen, Groningen, The Netherlands

**Keywords:** Contrast, Electron microscopy, Neodymium, Alternative contrast, Neodymium acetate, Uranyl acetate

## Abstract

Uranyl acetate is the standard contrasting agent in electron microscopy (EM), but it is toxic and radioactive. We reasoned neodymium acetate might substitute uranyl acetate as a contrasting agent, and we find that neodymium acetate indeed can replace uranyl acetate in several routine applications. Since neodymium acetate is not toxic, not radioactive and easy to use, we foresee neodymium will replace uranyl in many EM sample preparation applications worldwide.

## Introduction

Uranyl acetate has been the standard contrasting agent in transmission electron microscopy (TEM) for decades (Watson 1958a, b). However, its use is increasingly hampered by regulations by governments due to its radioactive properties as well as its high toxicity. Therefore, alternatives are being searched for, including lanthanides or platinum blue (Hosogi et al. 2015; Ikeda et al. 2011; Inaga et al. 2007; Yamaguchi et al. 2010) as well as the use of less defined substances such as oolong tea extract (Sato et al. 2008; He and Liu 2017). Despite these published alternatives, uranyl acetate (UAc) is still the standard for EM contrasting. We tested published lanthanides and commercial solutions as a replacement for UAc, but were not satisfied since these alternatives were prone to contamination and lead to variable results. In classical TEM, where area selection is done by the operator, recording artefacts may be circumvented by choosing contamination-free areas, but in combination with large-scale EM showing the whole section the presence of drying spots and precipitates will be recorded (Kuipers et al. 2015). Here the use of non-radioactive neodymium acetate as an alternative for uranyl acetate is advocated. In the periodic table the vertical ordering of elements in groups is based on the presence of the same number of electrons in their outermost shell, which determines their chemical and physical properties. Because neodymium (Nd) is right above uranium (U) we reasoned that the chemical properties of UAc and NdAc would be very similar in binding to tissue in ultrathin sections thus leading to a similar amount of contrast.

## Material and methods

Neodymium (III) acetate (Sigma Aldrich) was dissolved as a 4% solution in water by heating in a water bath at 60° for 10 min. After vortexing and cooling to room temperature the pale pink solution was spun in an Eppendorf centrifuge at maximal speed for 5 min before use. Uranyl acetate (Merck) was prepared as a 2% solution in water and spun as above before use.

Fresh cut 100 nm sections of rat pancreas in EPON or Durcupan (Ravelli et al. 2013) on formvar coated 1 mm single slot copper grids were contrasted on drops of NdAc or UAc for 20–60 min and subsequently washed on six drops of water, dried and examined in a FEI Cm100bio transmission electron microscope (TEM) operated at 80 kV. Imaging was done with exact the same beam spreading and fixed scaling in the OlympusSIS iTEM software controlling a Morada camera. Scaling was set with UAc as a reference and kept the same for NdAc and the non-stained sample. Quantification of contrast of NdAc, UAc and the none-stained sample was done by exporting the greyscale values from the histogram in ImageJ/Fiji and plot these values as an overlay line graph using Excel. These single contrasted sections were also scanned at 2.5 nm pixel size using a Zeiss Supra55 SEM with STEM detection using Fibics ATLAS software (Sokol et al. 2015). Data were exported as html and uploaded to a server (www.nanotomy.org). Other sections were contrasted with NdAc or UAc as above followed by 2 min Reynolds lead citrate.

In situ staining (en bloc staining) was done on U2OS cells or rat liver tissue (approx. 1 mm^3^) fixed with 2% GA + 2% PFA. After standard osmium post-fixation for 30 min at 4° (cells) or 2 h at 4° (tissue; Kuipers et al. 2015; Ravelli et al. 2013; Sokol et al. 2015), washing three times with water, staining was done with 2% UAc or 4% NdAc at room temperature (cells 30 min, tissue 30, 60 or 120 min). Samples were washed with water, dehydrated with ethanol series and embedded in EPON. 100 nm sections were cut and examined in a FEI Cm100bio TEM at 80 kV without further contrasting at fixed scaling as described above.

Negative staining was done on amyloid-β fibers (kind gift of L. Jansen, UMC Groningen) adhered to freshly prepared formvar film on 150 mesh copper grids for 15 min. Liquid was drained with a filter paper and grids were placed on a drop of staining solution for 1 min, washed by dipping in a drop of water, air dried and examined in the TEM at 80 kV.

For elemental detection 100 nm sections of rat pancreas in Durcupan were collected on formvar coated 1 mm single slot pyrolytic carbon grids (Agar Scientific; detailed in Scotuzzi et al. 2017). Elemental maps were collected with an Oxford X-Max 150 mm^2^ EDX detector mounted on a Zeiss Supra55 SEM operated at 10 kV in high current mode with a 120 µm aperture. X-ray data collection was done with process time 4 of 20 frames with pixel dwell time 50 µs at 1024 pixel resolution. The corresponding EM image was recorded using the secondary electron detector (SE2) at the same SEM settings.

## Results and discussion

Neodymium acetate (NdAc) is not soluble at high concentrations in water. Staining sections with a saturated solution showed speckles, likely precipitated NdAc. Therefore, solutions of 2, 4 and 8% NdAc were made and stainings were analyzed: The 4% and 8% samples resulted in equal better contrast compared to 2% NdAc staining (data not shown). Therefore, 4% NdAc was used in all further experiments. Solutions freshly prepared or 4 weeks stored at room temperature did not show any differences in contrast. Also no difference was seen between 20 min staining and 60 min staining. UAc was replaced for this NdAc solution to address its use as a generic stain in EM for (i) post staining of sections; (ii) en bloc (pre-embedding) contrasting and (iii) negative stain.

**Fig. 1:**
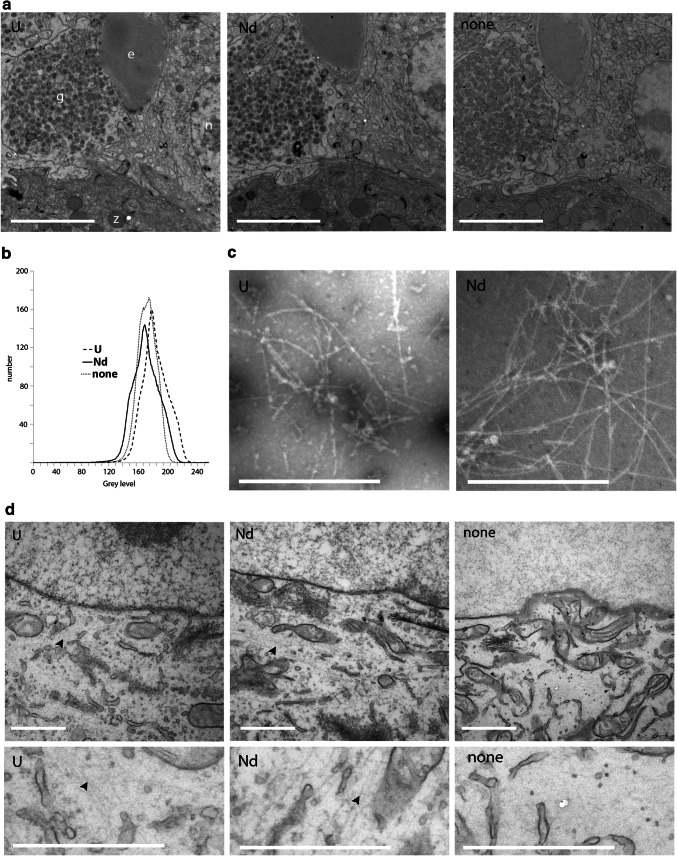
Neodymium acetate replacing uranyl acetate for contrasting EM samples. **a** TEM images of 100 nm sections rat pancreas embedded in EPON stained with either 2% uranyl acetate, 4% neodymium acetate or without any stain showing an increased contrast of UAc and NdAc compared to no staining. To visualize the difference in contrast images were recorded with same beam spreading and fixed scaling. *e* erythrocyte, *n* nucleus, *g* glucagon granules, *z* zymogen granules. Note that only the first panel is annotated, because the other two show the same structures. Bars: 5 µm. **b** Overlays of histograms of images in **a**, showing the distribution of grey values. NdAc (solid line) and UAc (dashed line) have more grey values, so more contrast, compared to a non-contrasted sample (dotted line). **c** Negative stain with NdAc or UAc show same structures and contrast applied to amyloid-β fiber ultrastructural examination. Bars: 0.5 µm. **d** In situ staining (en bloc staining) of U2OS cells with UAc or NdAc gives contrast to proteins in the cytoplasm as well as the nucleus compared to no staining where only osmium contrast is seen in mainly membranes. Note that Nd even stains cytoskeletal proteins more clearly compared to UrAc (arrowheads), which are not visible at all in the none contrasted sample. Bars 1 µm

**Fig. 2:**
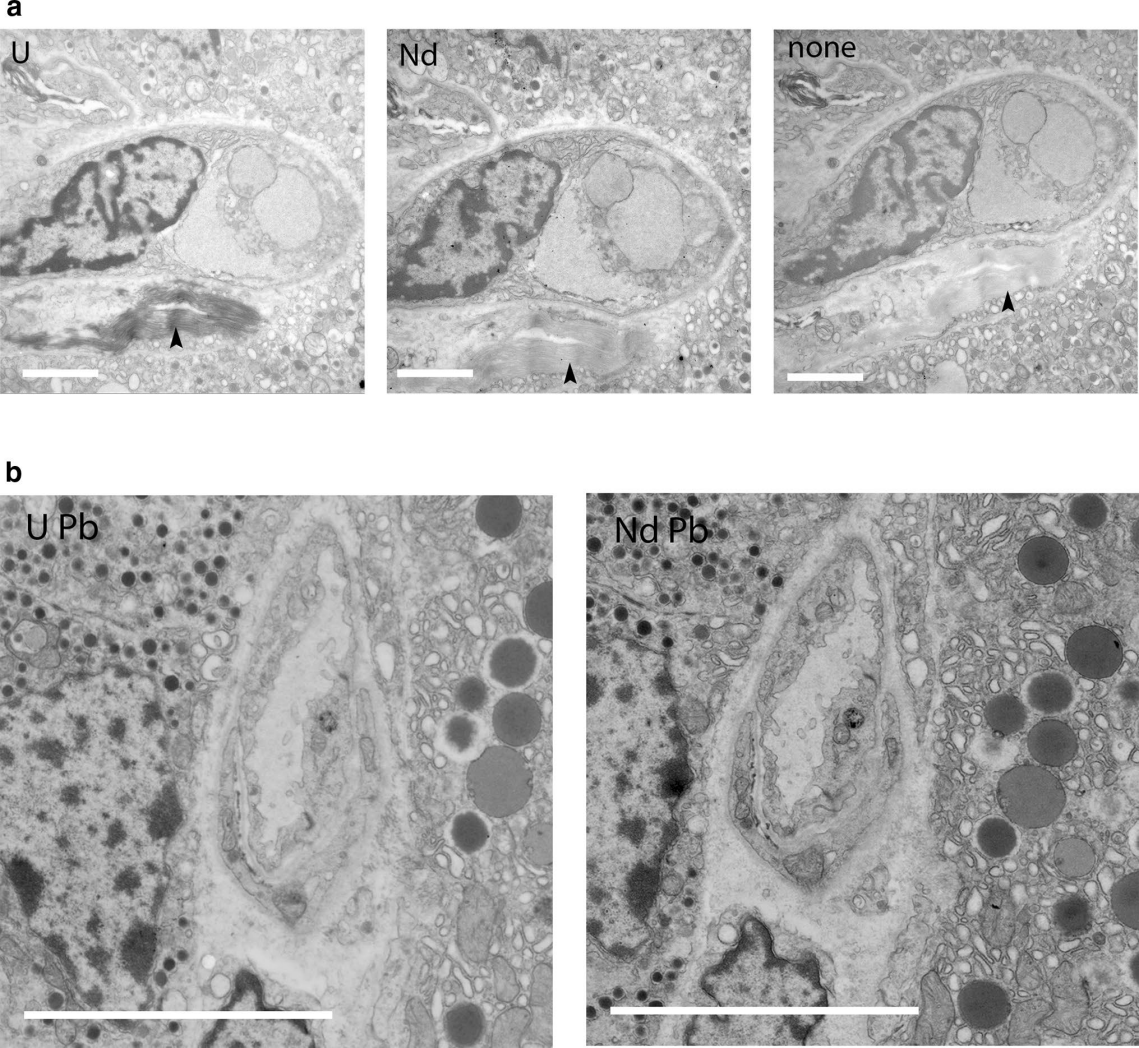
Neodymium Acetate replacing uranyl acetate in durcupan embedded sections, also combined with Reynolds lead citrate. **a** TEM images of 100 nm sections rat pancreas embedded in Durcupan stained with either 2% uranyl acetate, 4% neodymium acetate or without any stain showing an increased contrast of UAc and NdAc compared to no staining. To visualize the difference in contrast images were recorded with same beam spreading and fixed scaling. Note the equal contrast for UAc and NdAc, only collagen being more dark with UAc (arrow heads). Bars: 2 µm. **b** TEM images of 100 nm sections rat pancreas embedded in Durcupan stained with either 2% uranyl acetate, 4% neodymium acetate followed by Reynolds Lead Citrate contrasting give very similar contrast. Bars: 5 µm

The standard post staining of sections from aldehyde and osmium fixed tissue with 2% UAc or 4% NdAc stained the same cellular structures with a very similar contrast (Fig. 1a). Recording images with the same beam spreading and fixed scaling for the contrast setting of the camera enabled direct comparison of the contrast generated, where low contrast has less grey values resulting in a narrow histogram and more contrast in a broader histogram. From the overlay of the histograms of the total image from non-contrasted sample compared to the contrasted samples a clear increase in contrast can be seen (Fig. 1b) for both UAc as well as NdAc. This was also directly visible by the contrast generated on the phosphorescent screen of the TEM (data not shown): the Nd and UAc contrasted samples allowed to recognize cell types in the tissue making navigation easy, whereas the non-contrasted tissue was barely visible precluding proper navigation. The granules of the endocrine delta cell as well as the granules of the acinar cells have similar contrast (Fig. 1a). Also the erythrocyte shows similar contrast. Both NdAc and UAc stained the nucleus, but UAc seems to show some more contrast difference between heterochromatin and euchromatin. UAc also stained collagen fibers darker compared to NdAc (Fig. 2a). Since contrasting with UAc is mostly accompanied by staining with Reynolds lead citrate (Reynolds 1963), NdAc was combined with Reynolds lead staining. Rat pancreas staining with UAc plus lead or NdAc plus lead show very little differences in contrast (Fig. 2b). Again collagen is darker in UAc also with lead, but is still clearly recognizable in NdAc plus lead. A zoomable big dataset showing multiple cell types in a whole Islet of Langerhans plus exocrine tissue, ducts, blood capillaries can be found at www.nanotomy.org.

**Fig. 3:**
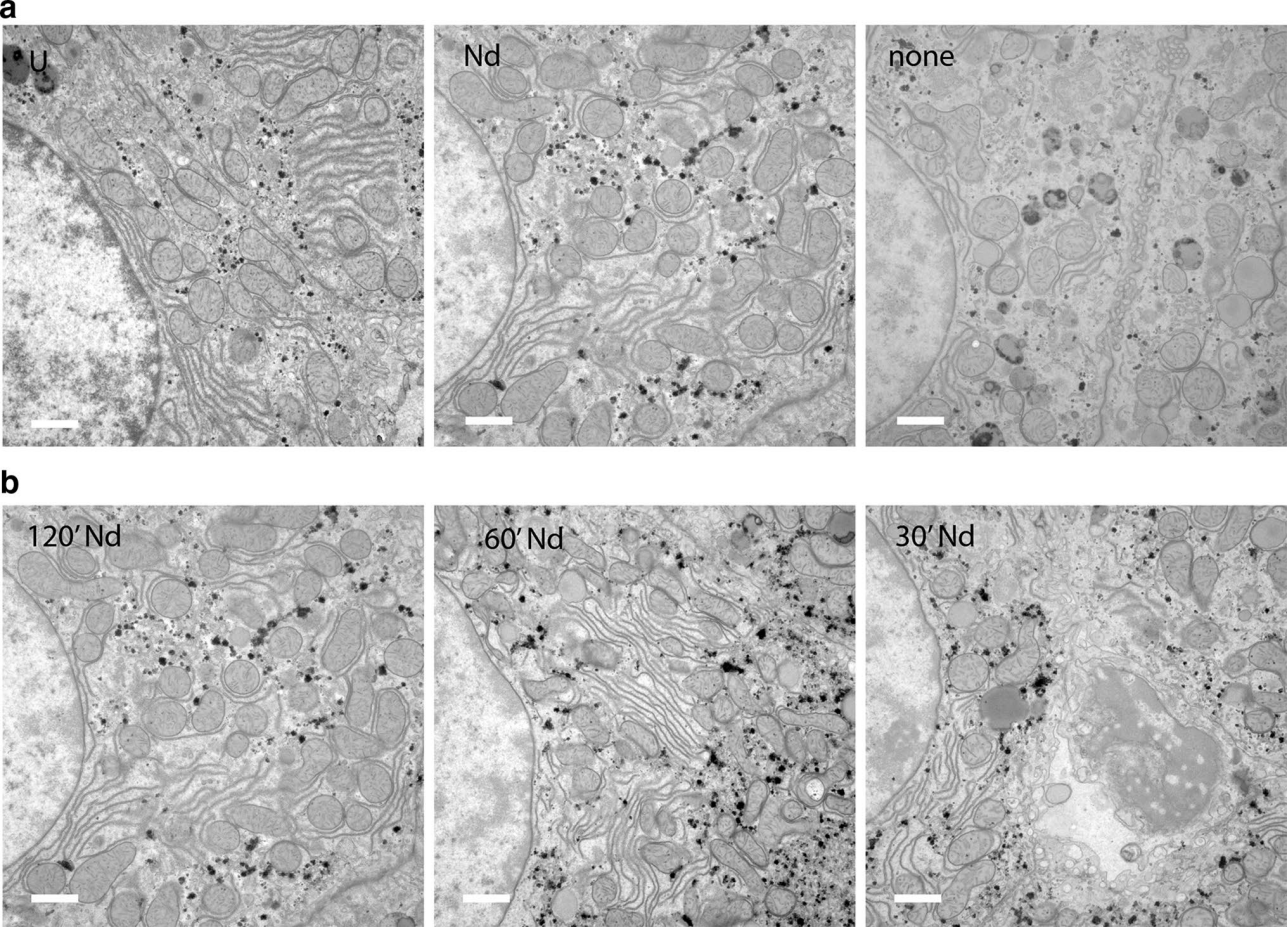
NdAc used in En Block staining of cells and tissue can replace UAc. **a** Detail of U2OS cells after In situ staining (en bloc staining) with UAc or NdAc. Note that Nd even stains cytoskeletal proteins more clearly compared to UrAc (arrowheads), which are not visible at all in the none contrasted sample. Bars 1 µm. **b** Rat liver tissue stained en bloc with NdAc replacing UAc without any other post staining on sections before imaging in TEM. NdAc gave contrast to proteins and DNA as does UAc, much like seen with NdAc staining on sections. Bars 1 µm. **c** Time series of en block staining using NdAc showing already good contrast after 30 min. Bars 1 µm

Samples fixed with Osmium and embedded in Durcupan show a higher contrast by itself, making the difference between non-stained and stained samples smaller. But also here NdAc gave similar contrast as UAc (Fig. 2a). NdAc turned out to be very effective as an in situ stain for staining of samples before plastic embedding, so called en-bloc staining. U2OS cells without NdAc or UAc only show membranes from Osmium contrast. Introducing NdAc or UAc yields very similar contrast to the nucleus and cytoplasm by staining proteins as can be seen by for example cytoskeletal proteins not visible in the osmium only cells (Fig. 1d). Also with en-bloc staining of rat liver tissue NdAc can replace UAc, although mitochondria appear a bit different (Fig. 3a), maybe caused by the proteins inside the mitochondria being more stained with Nd causing the osmium stained cristae membranes standing out less. The time for the en-bloc staining can be as short as 30 min, since no difference in contrast was seen between 120, 60 or 30 min incubation in 4% NdAc (Fig. 3b).

Negative stain is another application where UAc is widely used. Staining amyloid-β fibers with NdAc gave indeed a staining very similar to UAc stained fibers (Fig. 1c). Note that, depending on the sample, other negative stains may better replace UAc (De Carlo and Harris 2011).

**Fig. 4:**
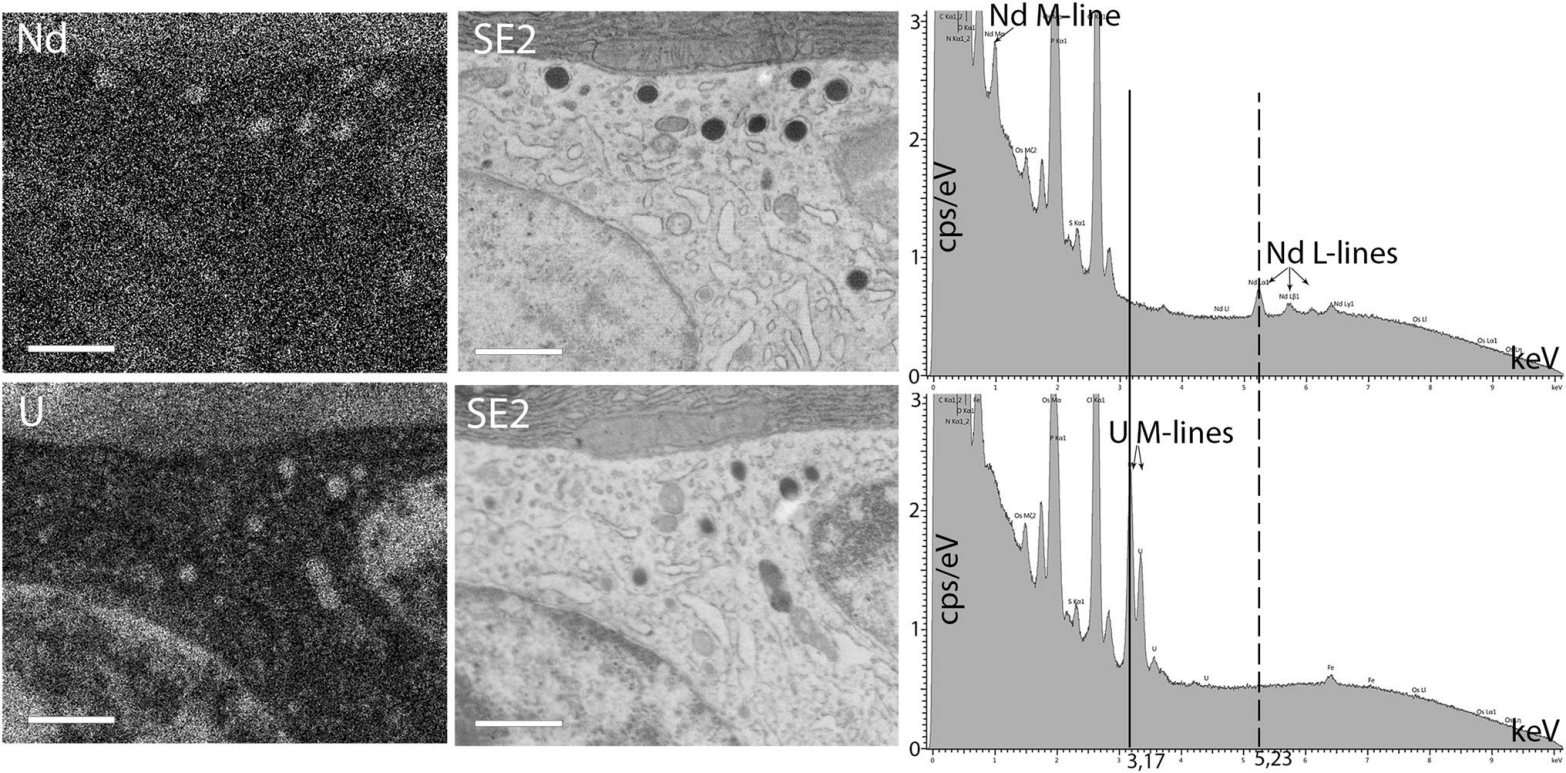
Neodymium is identified at the increased contrast regions of subcellular structures. Elemental analysis using EDX of an 100 nm section of rat pancreas reveals that Nd is present in the tissue after contrasting with a 4% NdAc solution as can be seen from the total sum X-ray energy spectrum. The localization of Nd in the NdAc stained section is similar to U in a UAc stained section, being the nuclear heterochromatin, the electron dense granules and the dense endoplasmic reticulum seen in the upper part of the image. Bars: 1 µm

To address whether the contrast seen in NdAc stained sections was indeed caused by the presence of Nd, we performed elemental analysis using elemental dispersive X-ray (EDX) analysis. EDX of NdAc stained pancreas shows that indeed Nd is localized in endocrine granules, heterochromatin of the nucleus and the dense ER network of the acinar cell. The same localization is seen for U when stained with UAc (Fig. 4). The image map for Nd is more noisy compared to U, which is caused by the higher Z number of U, so generating more x-rays. This is also reflected by higher peaks in the total sum spectrum for U versus Nd. Mapping of the Nd with either the L lines or the M lines gave exactly the same Nd image maps and the L lines were used to generate the map in Fig. 4.

We conclude that NdAc can replace UAc for most of its applications, as standard post sectioning counterstain, as an in-situ stain and as a negative stain. NdAc gives good contrast comparable with UAc using a camera in a transmission electron microscope, using secondary electron detection in a scanning electron microscope, as well as using a scanning transmission detector (STEM) in a scanning electron microscope. A methanol or acetone solution of UAc is also used in freeze substitution embedding after high pressure freezing. NdAc, however, is hardly soluble in these solvents, so probably cannot be used in freeze substitution embedding of high pressure frozen samples. Due to its low toxicity and low price we replace UAc for NdAc in most EM applications and foresee this will be widely implemented in EM contrasting.
